# Impact of an energy-conserving strategy on succinate production under weak acidic and anaerobic conditions in *Enterobacter aerogenes*

**DOI:** 10.1186/s12934-015-0269-6

**Published:** 2015-06-11

**Authors:** Yoshinori Tajima, Yoko Yamamoto, Keita Fukui, Yousuke Nishio, Kenichi Hashiguchi, Yoshihiro Usuda, Koji Sode

**Affiliations:** Institute for Innovation, Ajinomoto Co., Inc., 1-1 Suzuki-cho, Kawasaki-ku, Kawasaki, 210-8681 Japan; Research Institute for Bioscience Product & Fine Chemicals, Ajinomoto Co., Inc., 1-1 Suzuki-cho, Kawasaki-ku, Kawasaki, Japan; Department of Biotechnology, Graduate School of Engineering, Tokyo University of Agriculture and Technology, Naka-cho, Koganei, Tokyo Japan

**Keywords:** *Enterobacter aerogenes*, Succinate fermentation, Phosphoenolpyruvate carboxykinase, Glucose-PTS, Weak acidity, Anaerobic metabolism

## Abstract

**Background:**

Succinate is an important C4 building block chemical, and its production via fermentative processes in bacteria has many practical applications in the biotechnology field. One of the major goals of optimizing the bacterium-based succinate production process is to lower the culture pH from the current neutral conditions, as this would reduce total production costs. In our previous studies, we selected *Enterobacter aerogenes*, a rapid glucose assimilator at pH 5.0, in order to construct a metabolically engineered strain that could produce succinate under weakly acidic conditions. This engineered strain produced succinate from glucose with a 72.7% (g/g) yield at pH 5.7, with a volumetric productivity of 0.23 g/L/h. Although this demonstrates proof-of-concept that bacterium-based succinate fermentation can be improved under weakly acidic conditions, several parameters still required further optimization.

**Results:**

In this study, we genetically modified an *E. aerogenes* strain previously developed in our laboratory in order to increase the production of ATP during succinate synthesis, as we inferred that this would positively impact succinate biosynthesis. This led to the development of the ES08Δ*ptsG* strain, which contains the following modifications: chromosomally expressed *Actinobacillus succinogenes* phosphoenolpyruvate carboxykinase, enhanced fumarate reductase, inactivated pyruvate formate lyase, pyruvate oxidase, and glucose-phosphotransferase permease (enzyme IIBC^Glc^). This strain produced 55.4 g/L succinate from glucose, with 1.8 g/L acetate as the major byproduct at pH 5.7 and anaerobic conditions. The succinate yield and volumetric productivity of this strain were 86.8% and 0.92 g/L/h, respectively.

**Conclusions:**

Focusing on increasing net ATP production during succinate synthesis leads to increased succinate yield and volumetric productivity in *E. aerogenes*. We propose that the metabolically engineered *E. aerogenes* ES08Δ*ptsG* strain, which effectively produces succinate under weakly acidic and anaerobic conditions, has potential utility for economical succinate production.

## Background

Succinate is an important building block for the synthesis of many basic general chemicals such as γ-butyrolactone, tetrahydrofuran, and 1,4-butanediol. One specific example of its usage is the polymerization of succinate and 1,4-butanediol to produce polybutylene succinate polymer (PBS), which is a biodegradable plastic [1, 2]. The US Department of Energy (DOE) has recognized succinate as one of the top 12 building block chemicals produced from biomass via biological or chemical conversion [3]. Currently, succinate is mainly produced from maleic anhydride in petroleum chemical processes, and its worldwide usage is estimated at approximately 20–30 kton/year [4]. However, mid- and long-term risks of increases in oil price and increasing awareness of environmental pollution have generated interest in the microbial production of succinate from renewable resources [5, 6]. Several companies and joint ventures such as Myriant, BioAmber/Mitsui, Succinity (BASF/Corbion-Purac), and Reverdia (DSM/Roquette) have initiated large-scale fermentative production of succinate [7].

For the last several decades, a number of groups have successfully achieved bacterium-based succinate fermentation using engineered strains of *Escherichia coli*, *Corynebacterium glutamicum*, *Actinobacillus succinogenes*, *Anaerobiospirillum succiniciproducens*, and *Mannheimia succiniciproducens* [8–12]. Although all of these bacteria produced succinate effectively at neutral pH, this was not the case in acidic conditions [13–15]. This is because these bacteria are inherently unable to grow and assimilate carbon sources effectively under acidic conditions [16, 17]. Lowering the culture pH of succinate fermentation can reduce total the costs of succinate production by reducing the amount of alkali and acid used in the fermentation and recovery process [18, 19]. However, bacterial strains that can effectively produce succinate under acidic conditions have not been reported. Thus, there is a need to improve the economic and ecological impact of such bacteria in relation with the succinate production process.

To evaluate the potential of bacterium-based succinate fermentation under acidic conditions, we based our new platform on *Enterobacter aerogenes*, which can rapidly assimilate glucose at pH 5.0, and then developed the ES04/PCK+PYC strain in which by-product pathways are eliminated and heterogeneous carboxylation enzymes are introduced [20, 21]. In our first trial, we set a target pH value at 5.7, which means that we can reduce the amount of alkali consumed during succinate fermentation at pH 7.0 by more than 20%; this is according to calculations of the ionized form of succinate based on p*Ka* values (p*Ka*_1_ 4.16 and p*Ka*_2_ 5.6) [7]. This strain produced succinate from glucose with a 72.7% yield and a volumetric productivity of 0.23 g/L/h at pH 5.7 and anaerobic conditions [21]. However, these values are still lower than those obtained under neutral conditions using *E. coli* KJ122 and *C. glutamicum* Δ*ldhA*-pCRA717 [11, 12]. Additionally our results also show that volumetric productivity at pH 5.7 (0.23 g/L/h) was approximately half of that at pH 7.0 (0.47 g/L/h). Thus, lowering culture pH has a negative impact on succinate production in *E. aerogenes*; this has also been observed using *E. coli* [15]. There are two main elements that can be adjusted in order to improve succinate productivity under acidic conditions. The first is to use a general metabolic engineering approach in order to increase carbon flux toward the succinate synthesis pathway. However, an equally important adjustment is to increase the adaptability and acid-tolerance of the strains used under these conditions. In *E. coli*, acid tolerance mechanisms have been well studied, and its mechanism is conserved in *E. aerogenes* based on genome sequence analysis [22]. Under acidic conditions, the majority of ATP will be consumed for proton and anion efflux and other acid resistance mechanisms for pH homeostasis [16, 17]. In this regard, we speculated that energy demand for pH homeostasis under acidic conditions is likely higher than at neutral conditions [23]. Therefore, there is a higher risk of decreasing succinate production due to energy starvation under acidic and anaerobic conditions. For this reason, we focused on genetic modifications that would increase the ATP supply during succinate synthesis using a so-called ‘energy-conserving strategy’ that was previously established in *E. coli* [24, 25]. In this study, we applied this strategy in *E. aerogenes* and evaluated its impact on succinate production under weakly acidic and anaerobic conditions.

To construct an energy-conserving succinate synthesis pathway in *E. aerogenes*, we introduced ATP-forming phosphoenolpyruvate carboxykinase (PCK) derived from *Actinobacillus succinogenes* [26], and then disrupted the genes involved in the glucose phosphotransferase system (glucose-PTS). This novel ES08Δ*ptsG* strain produced 55.4 g/L succinate from glucose, with an 86.8% yield for 60 h at pH 5.7 and anaerobic conditions. Thus, this approach can be applied to *E. aerogenes*, in order to successfully increase succinate production.

## Results

### Strategy for increasing net ATP production during succinate synthesis in *E. aerogenes*

**Figure 1:**
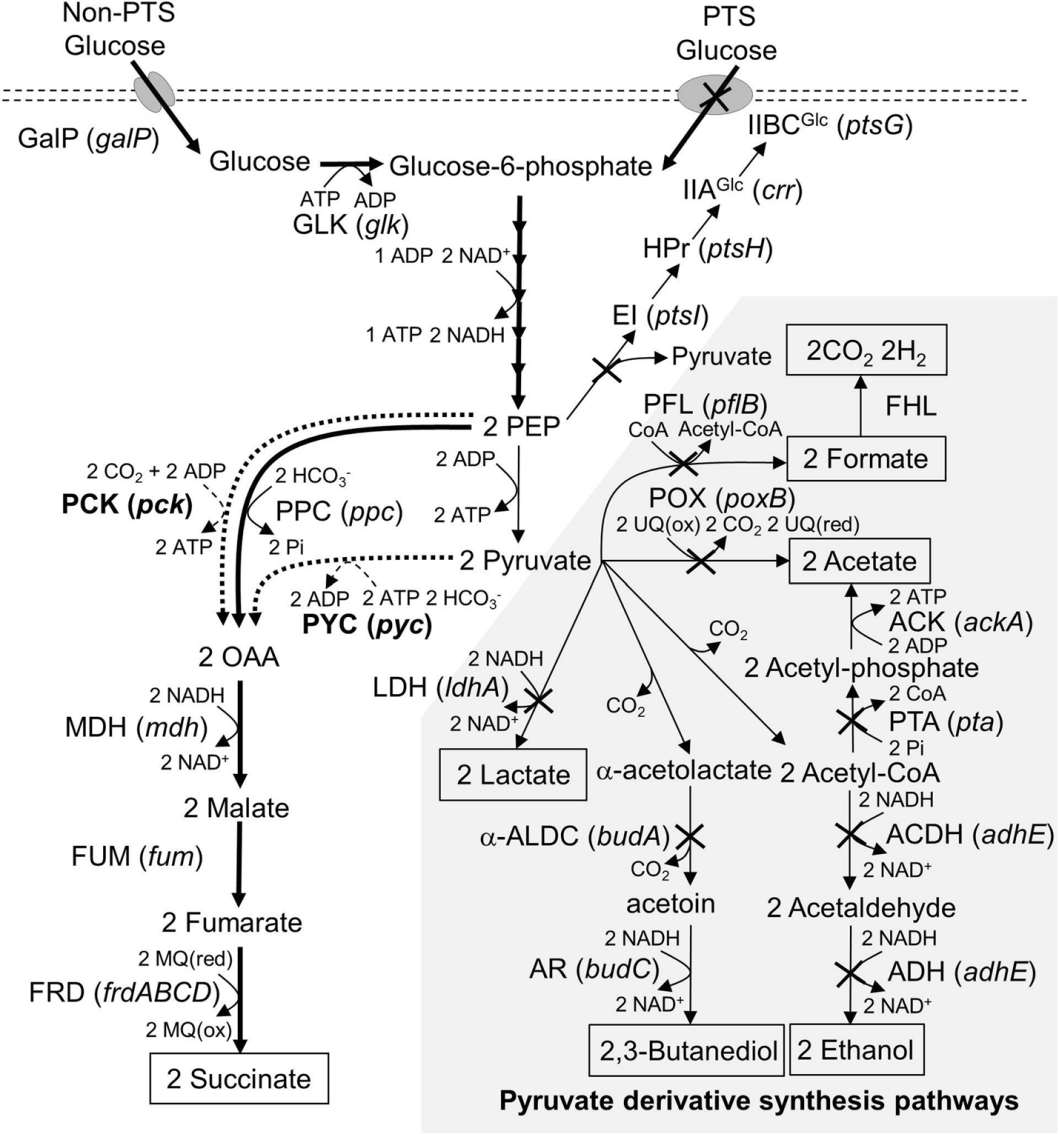
Pathways involved in ethanol, 2, 3-butanediol, lactate, acetate, formate (*thin arrows*), and succinate synthesis (*thick arrows*) in *E. aerogenes. Broken arrows* indicate exogenous pyruvate carboxylase and PEP carboxykinase from *C. glutamicum* and *A. succinogenes*, respectively. The gene names or locus symbols are shown in *parentheses*. *PCK* PEP carboxykinase, *PYC* pyruvate carboxylase, *PPC* PEP carboxylase, *MDH* malate dehydrogenase, *FUM* fumarase, *FRD* fumarate reductase, *LDH* d-lactate dehydrogenase, *α-ALS* α-acetolactate synthase, *α-ALDC* α-acetolactate decarboxylase, *AR* acetoin reductase, *ACDH* acetaldehyde dehydrogenase, *ADH* alcohol dehydrogenase, *PTA* phosphate acetyltransferase, *ACK* acetate kinase, *POX* pyruvate oxidase, *PFL* pyruvate formate lyase, *FHL* formate hydrogen lyase, *EI* enzyme I, *HPr* histidine-containing phosphocarrier protein, *IIA* ^*Glc*^ glucose-specific IIA component, *IIBC* ^*Glc*^ glucose-PTS permease, *GalP* galactose/proton symporter, *GLK* glucokinase.

**Figure 2:**
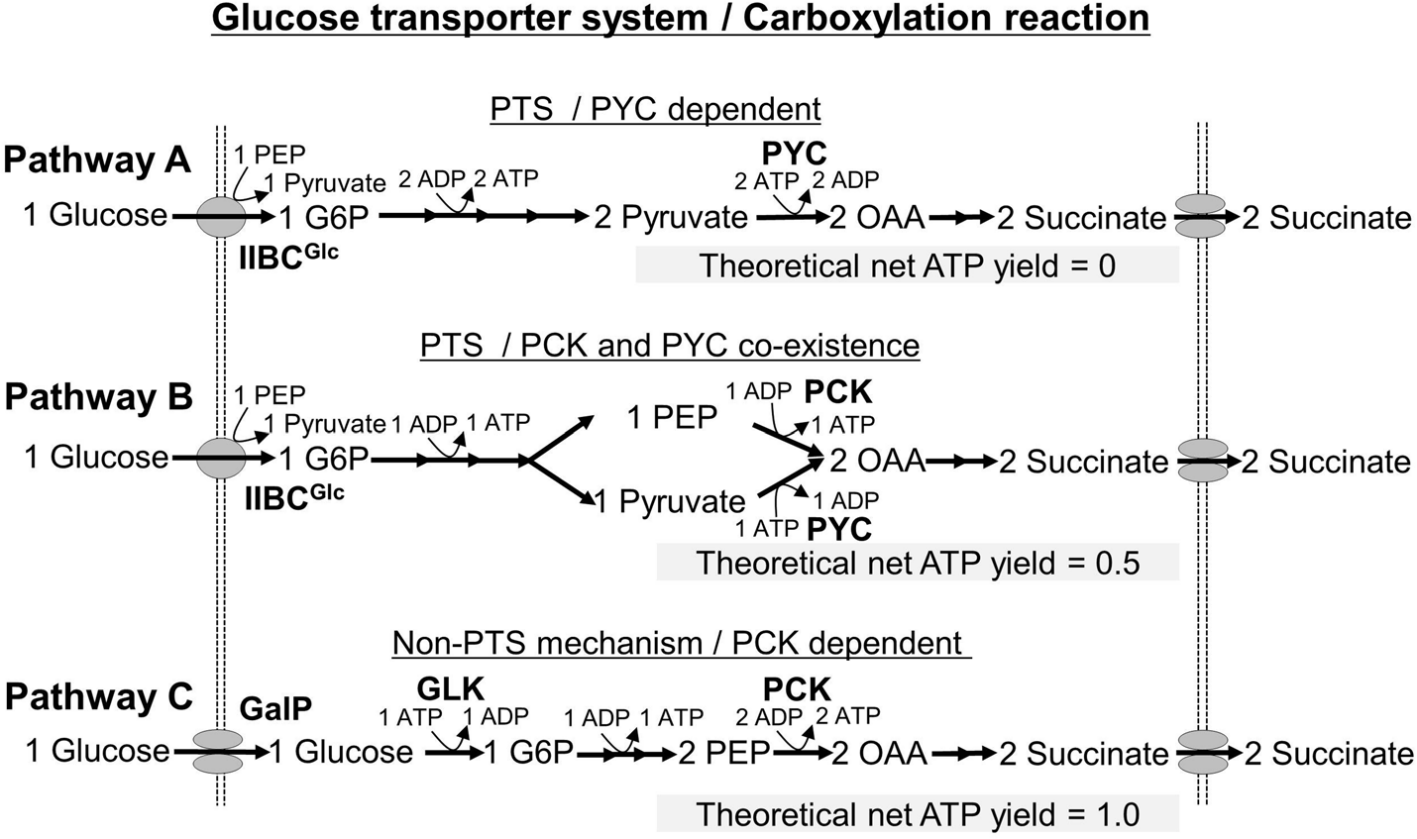
Theoretical net ATP yields from various pathways of succinate synthesis. Theoretical net ATP yield (mol ATP gained per mol of succinate produced) is estimated in all pathways when two moles of succinate are generated from one mole of glucose. (a) Pathway A, in which succinate is synthesized via glucose-PTS and PYC-dependent carboxylation reactions, (b) pathway B, which uses glucose-PTS and PYC+PCK co-dependent carboxylation reactions, (c) pathway C, which uses non-PTS and PCK-dependent carboxylation reactions.

To realize an energy conserving strategy that would increase net ATP production during succinate synthesis in *E. aerogenes*, we focused on modifications of carboxylation enzymes and the glucose uptake system (Figure 1). The glucose-PTS is the dominant means by which glucose is imported in *Enterobacteriaceae* such as *E. coli* [27]. In the first step of the phosphorylation cascade in this system, enzyme I (EI) encoded by *ptsI* is phosphorylated, which leads to dephosphorylation of PEP and formation of pyruvate (Figure 1). As a result, one mole of pyruvate is theoretically produced when one mole of glucose is imported into the cell (Figure 2, pathway A). To direct pyruvate generated via the glucose-PTS to oxaloacetate (OAA), pyruvate carboxylase (PYC) has been widely used in *E. coli* and *C. glutamicum* [28–30]. If PYC serves as the dominant carboxylation reaction, two moles of pyruvate are converted to two moles of OAA, with two moles of ATP consumption. Finally, two moles of succinate are generated from one mole of glucose. When succinate is synthesized by this route, the demand and supply of ATP are balanced. The theoretical net ATP yield (obtained as moles ATP produced per mole of succinate) as a result of using this route is 0 (Figure 2, pathway A).

Previously, we developed the *E. aerogenes* ES04/PYC+PCK strain, which expresses two heterogeneous carboxylation enzymes, *C. glutamicum* PYC and *A. succinogenes* PCK (Table 1) [21]. In this situation, one mole of PEP is converted to one mole of OAA via a PCK-dependent reaction, and one mole of ATP is generated [26], while one mole of pyruvate derived from the glucose-PTS is converted to one mole of OAA via a PYC-dependent reaction, and one mole of ATP is consumed (Figure 2, pathway B). As a result, the theoretical net ATP yield is 0.5, indicating that the utilization of this route generates extra ATP.

**Table 1:** Microbial strains and plasmids used in this study

Strains and plasmids	Description	Antibiotic resistance	References or sources
Strain			
AJ110637	Isolated wild-type strain (FERM BP-10955)	–	[20]
ES04	AJ110637 Δ*adhE* Δ*ldhA* Δ*pta* Δ*budA*	Km	[21]
ES04/PCK	AJ110637 Δ*adhE* Δ*ldhA* Δ*pta* Δ*budA* harboring pSTV28-*pck*	Km, Cm	[21]
ES04/PCK + PYC	AJ110637 Δ*adhE* Δ*ldhA* Δ*pta* Δ*budA* harboring pSTV28-*pck* + *pyc*	Km, Cm	[21]
ES05	AJ110637 Δ*adhE* Δ*ldhA* Δ*pta* Δ*budA* Δ*poxB*	Km	This work
ES05/PCK	AJ110637 Δ*adhE* Δ*ldhA* Δ*pta* Δ*budA* Δ*poxB* harboring pSTV28-*pck*	Km, Cm	This work
ES06	AJ110637 Δ*adhE* Δ*ldhA* Δ*pta* Δ*budA* Δ*poxB*::*P*_*tac*_-*pck* (*A. succinogenes*)	Km	This work
ES07	AJ110637 Δ*adhE* Δ*ldhA* Δ*pta* Δ*budA* Δ*poxB*::*P*_*tac*_-*pck* (*A. succinogenes*) *P*_*tac*_-*frdABCD*	Km	This work
ES08	AJ110637 Δ*adhE* Δ*ldhA* Δ*pta* Δ*budA* Δ*poxB*::*P*_*tac*_-*pck* (*A. succinogenes*) *P*_*tac*_-*frdABCD* Δ*pflB*	Km	This work
ES08Δ*ptsG*	AJ110637 Δ*adhE* Δ*ldhA* Δ*pta* Δ*budA* Δ*poxB*::*P*_*tac*_-*pck* (*A. succinogenes*) *P*_*tac*_-*frdABCD* Δ*pflB* Δ*ptsG*	Km	This work
ES08Δ*ptsI*	AJ110637 Δ*adhE* Δ*ldhA* Δ*pta* Δ*budA* Δ*poxB*::*P*_*tac*_-*pck* (*A. succinogenes*) *P*_*tac*_-*frdABCD* Δ*pflB* Δ*ptsI*	Km	This work
Plasmid			
pRSFRedTER	Broad-host-range λ Red-expressing plasmid	Cm	[44]
pMW- *attL*_λ_-Km^R^- *attR*_λ_	Cassette for gene disruption containing kanamycin resistance gene	Km	[45]
pRSF-*P*_*ara*_-IX	Plasmid for removal of antibiotic resistance genes	Cm	[21]
pSTV28	Plasmid vector with a replication origin of pACYC184	Cm	TAKARA BIO
pSTV28-*pck*	Plasmid for expression of *pck* from *A. succinogenes*	Cm	[20]
pSTV28-*pck*+*pyc*	Plasmid for co-expression of *pck* from *A. succinogenes* and *pyc* from *C. glutamicum*	Cm	[21]

A maximum theoretical net ATP yield is achieved by a combination of the non-PTS mechanism and a PCK-dependent carboxylation pathway (Figure 2, pathway C). In order to increase the intracellular PEP level and thus maximize carbon flux for ATP-forming PCK reactions, it has been proposed that the glucose-PTS in *E. coli* could be replaced with a non-PTS mechanism [24, 25]. The non-PTS mechanism is comprised of sugar-proton symporters and glucokinase. GalP is a member of the sugar-proton symporter family, and mainly mediates glucose uptake in glucose-PTS deficient *E. coli* mutants [31]. Glucose imported via GalP is phosphorylated and converted to glucose-6-phosphate (G6P) in an ATP-dependent manner by glucokinase (GLK) (Figure 1). In this system, one mole of glucose is imported via GalP, and is then converted to G6P; one mole of ATP is consumed by GLK during this reaction. Two moles of PEP are produced from one mole of glucose without ATP generation. All the PEP is converted to OAA via a PCK-dependent reaction, which is associated with ATP generation (Figure 2, pathway C). As a result, the theoretical net ATP yield is maximized and reaches 1.0, which is twofold higher than that in the case of pathway B described above. According to this pathway, we therefore introduced *A. succinogenes* PCK, and an inactivated glucose-PTS, to the ES04 strain (Table 1).

### Inactivation of pyruvate oxidase in ES04

**Figure 3:**
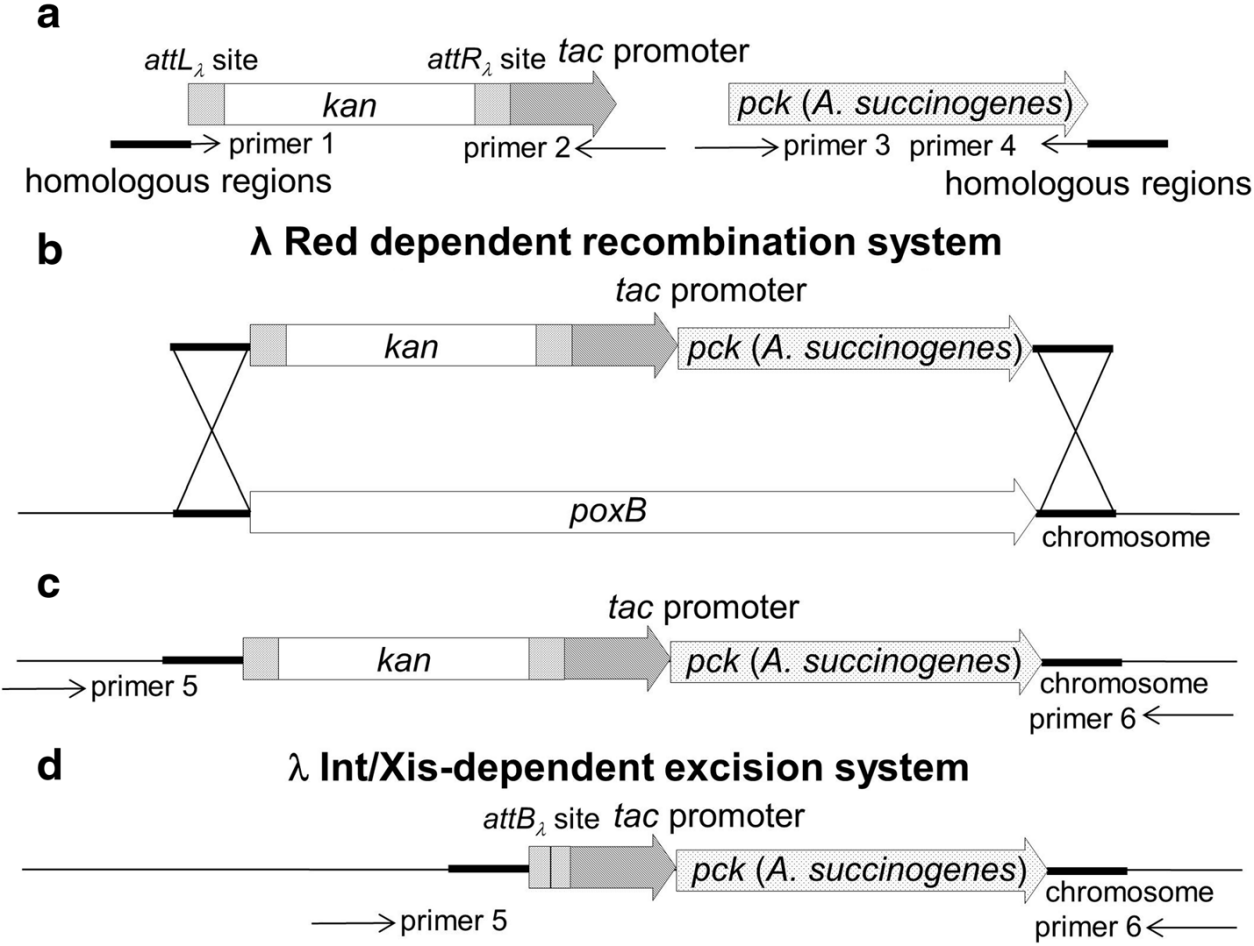
Scheme of *A. succinogenes* PCK expression cassette integration into the *poxB* locus using the λ Red- and Int/Xis-dependent systems. **a** Construction of *A. succinogenes* PCK expression cassette, **b** introduction of the PCK expression cassette into *poxB* locus on chromosome by λ Red-dependent recombination system using pRSFRedTER, **c** replacement of the *poxB* gene of the ES06 strain with the PCK expression cassette, and **d** removal of the kanamycin resistance gene from chromosome by the λ Int/Xis-dependent excision system using pRSF-*P*_*ara*_-IX. These genetic manipulations leave a 31-bp *attB*_*λ*_ site (5′-TGAAGCCTGC TTTTTTATAC TAACTTGAGC G-3′) in the chromosome.

The ES04 strain produced acetate, a major by-product that accumulated to 1.4 g/L, resulting in a 17.3% yield (Table 2). For reducing acetate formation in this strain, we selected an open reading frame of the *poxB* gene encoding pyruvate oxidase (POX) as the target locus for integration of an *A. succinogenes* PCK expression cassette (Figure 3). POX is a flavoprotein dehydrogenase located on the peripheral membrane. It catalyzes the decarboxylation of pyruvate to form acetate and CO_2_, coupled to ubiquinone reduction in *E. coli* (Figure 1) [32]. Targeted inactivation of POX has been used to reduce the acetate production and to improve succinate production in *E. coli* [11]. Before integration of the PCK expression cassette at the *poxB* gene locus, we investigated the effect of *poxB* inactivation on succinate production in the ES04 strain. A Δ*poxB* mutant was obtained from the ES04 strain, and then designated as the ES05 strain (Table 1). The PCK expression plasmid, pSTV28-*pck*, was introduced into both the ES04 and ES05 strains to enhance succinate production (Table 1). Succinate productivity of these strains under weakly acidic conditions was then evaluated. The ES04/PCK and ES05/PCK strains produced 6.4 and 6.8 g/L succinate from 10.4 and 11.0 g/L of glucose, resulting in 61.5 and 61.8% succinate yields, respectively (Table 2). The acetate titer and yield in ES05/PCK were 0.6 g/L and 5.4%, which is slightly lower when compared with those in ES04/PCK (0.8 g/L, corresponding to a 7.7% yield). These results indicated that inactivation of POX in the ES04/PCK strain slightly reduced acetate production, but had a negligible impact on succinate production.

**Table 2:** Parameter profiles in 1.5-mL-scale microfuge tube succinate fermentation reactions

Strain	End-pH	Biomass^a^, g[DCW]/L	Consumed glucose, g/L	End-product, g/L						Succinate Yield^b^, % (g/g)
				Pyruvate	Lactate	Malate	Succinate	Formate	Acetate	
ES04^c^	5.8 ± 0.1	5.2 ± 0.3^d^	8.1 ± 0.6	0.8 ± 0.1	<0.1	<0.1	1.6 ± 0.1	0.6 ± 0.1	1.4 ± 0.1	19.7
ES04/PCK	5.6 ± 0.1	5.7 ± 0.3	10.4 ± 0.6	0.4 ± 0.1	<0.1	0.4 ± 0.1	6.4 ± 0.1	0.3 ± 0.1	0.8 ± 0.1	61.5
ES05	5.8 ± 0.1	5.2 ± 0.3	7.4 ± 0.4	1.1 ± 0.2	<0.1	<0.1	1.6 ± 0.1	0.7 ± 0.1	0.9 ± 0.1	21.6
ES05/PCK	5.6 ± 0.1	5.7 ± 0.3	11.0 ± 0.6	0.5 ± 0.1	<0.1	0.4 ± 0.1	6.8 ± 0.1	0.3 ± 0.1	0.6 ± 0.1	61.8
ES06	5.5 ± 0.1	5.8 ± 0.3	12.0 ± 0.4	1.0 ± 0.1	<0.1	0.5 ± 0.1	7.6 ± 0.4	0.3 ± 0.1	0.6 ± 0.1	63.3
ES07	5.5 ± 0.1	5.9 ± 0.3	14.2 ± 0.8	1.0 ± 0.1	<0.1	0.2 ± 0.1	9.1 ± 0.3	0.4 ± 0.1	0.5 ± 0.1	64.1
ES08	5.5 ± 0.1	5.8 ± 0.3	14.5 ± 0.8	1.0 ± 0.1	<0.1	0.2 ± 0.1	9.2 ± 0.3	0.2 ± 0.1	0.5 ± 0.1	63.4
ES08Δ*ptsG*	5.7 ± 0.1	5.4 ± 0.4	11.5 ± 0.3	0.6 ± 0.1	<0.1	0.1 ± 0.1	8.8 ± 0.2	0.2 ± 0.1	0.7 ± 0.1	76.5
ES08Δ*ptsI*	5.9 ± 0.1	5.3 ± 0.3	6.2 ± 0.8	0.3 ± 0.1	ND	0.1 ± 0.1	5.4 ± 0.3	<0.1	0.3 ± 0.1	87.1

*ND* not determined.

^a^Initial biomass was adjusted to ~5.0 g[DCW]/L.

^b^Succinate yield is grams of product per grams of consumed glucose expressed as a percentage.

^c^All the mutants were cultivated for 24 h.

^d^Data are expressed as mean ± SD of four independent experiments.

### Integration of an *A. succinogenes* PCK expression cassette at the *poxB* locus of the ES04 strain

**Figure 4:**
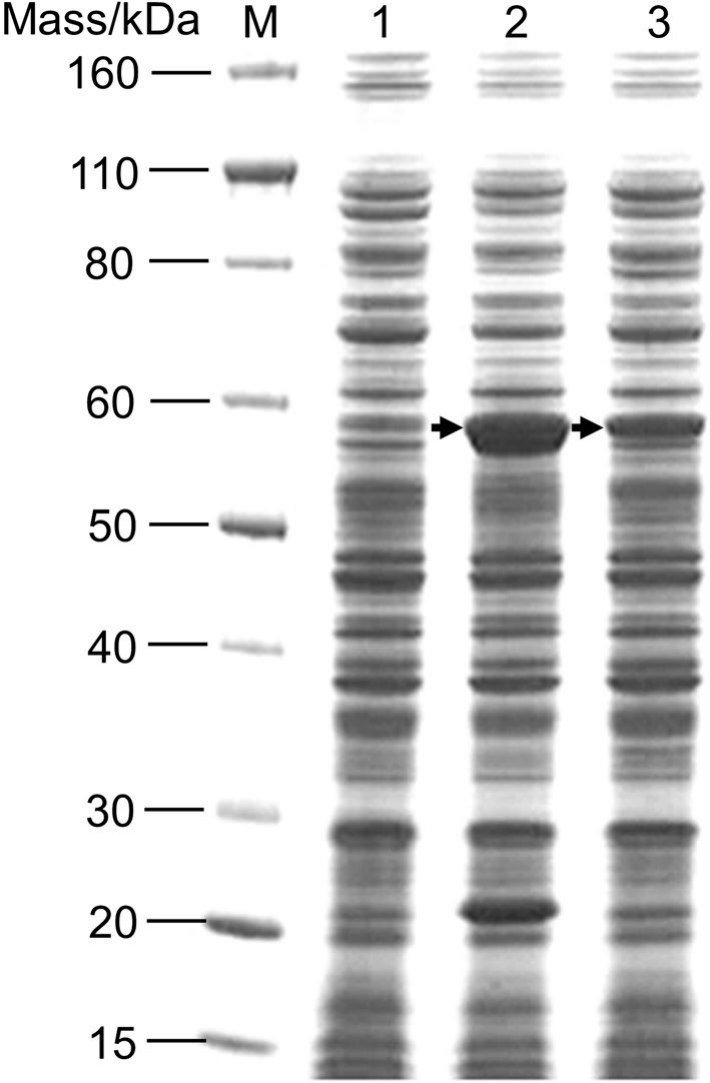
SDS-PAGE of *A. succinogenes* PCK. Soluble fractions were prepared from ES05, ES05/PCK, and ES06 grown under aerobic conditions. *1* ES05; *2* ES05/PCK; *3* ES06; *M* protein standard. *Arrows* indicate *A. succinogenes* PCK (59 Mass/kDa). Ten micrograms of protein was applied per *lane*.

The *A. succinogenes* PCK expression cassette was introduced into the *poxB* locus in the ES04 strain. This new strain was designated as ES06 (Table 1). To determine the level of PCK expression in ES06, sodium dodecyl sulfate-polyacrylamide gel electrophoresis (SDS-PAGE) analysis was performed. As shown in Figure 4, a band of the predicted molecular weight of PCK was detected in a sample from ES06. These data show that the *tac* promoter is a robust driver of expression in *E. aerogenes*, as was observed in *E. coli*. In addition, we found that the level of PCK expression in ES06 is lower than observed with plasmid-based expression in the ES05/PCK strain (Figure 4).

In order to determine how the level of PCK expression impacted succinate production, we compared the ES06 strain with the reference strain, ES05/PCK. As shown in Table 2, the succinate titer in ES06 (7.6 g/L) was 1.1 times higher than that in ES05/PCK (6.8 g/L), whereas the succinate yield was approximately 60% in both cases. It was suggested that the difference in succinate titer between these two strains could be explained by the differences in intracellular PEP levels. This was because the PEP level specifically affected glucose uptake due to control of the phosphorylation status of enzyme IIA^Glc^ [33, 34]. The PCK reaction competes with the PTS, because PEP is a common substrate in each case (Figure 1). Increased PCK activity may lead to a reduction of intracellular PEP levels, which in turn reduces the rate of glucose uptake. There is a lower level of PCK expression in ES06 compared with ES05/PCK; this favors the production of succinate, because the rate of glucose uptake was increased (Figure 4).

### Replacement of the *frdABCD* operon promoter with that of the *tac* promoter in the ES06 strain

Introduction of PCK also stimulated malate production, which reached 0.5 g/L in ES06 (Table 2). Malate is an intermediate metabolite of the reductive TCA cycle, which is linked to succinate synthesis (Figure 1). These data suggested that a rate limiting reaction for succinate production existed among reactions from malate to succinate, such as fumarase (FUM), fumarate reductase (FRD), and the succinate exporter (Figure 1). Among these, we speculated that FRD activity in particular might limit succinate production. FRD catalyzes the reduction of fumarate to succinate (Figure 1). This reaction is known as “fumarate respiration” and is required for maintenance of redox balance under anaerobic conditions [35].

In order to enhance FRD expression in ES06, the endogenous *frd*ABCD operon promoter region was replaced with the *tac* promoter, and the resulting strain was named ES07. As shown in Table 2, ES07 produced 9.1 g/L succinate from 14.2 g/L glucose, which is equivalent to a 64.1% succinate yield. The succinate titer in ES07 was approximately 1.2 times higher than that measured in the ES06 strain. In addition, malate titer and yield in ES07 (0.2 g/L with a yield of 1.4%) were reduced to approximately one-third of the corresponding values in the ES06 strain (0.5 g/L with a yield of 4.2%). These results suggested that FRD activity limited succinate production in the ES06 strain. Previous studies have shown that enhancement of malate dehydrogenase (MDH) activity led to increase succinate production in *E. coli* [36]. However, FRD-dependent reactions in reductive TCA also regulate succinate production in *E. aerogenes*, and therefore enhancing FRD enzyme activity can increase succinate production due to an improved redox balance.

### Effect of pyruvate formate lyase inactivation on succinate production

*Enterobacter aerogenes* is a model bacterium for the study of hydrogen production [37, 38]. In hydrogen synthesis, pyruvate formate lyase (PFL) converts pyruvate to formate and acetyl-CoA; formate can then be converted to hydrogen and carbon dioxide by formate hydrogen lyase (FHL) (Figure 1). *E. coli* PFL is a homodimeric protein in which the two subunits, PFL activating enzyme I and formate acetyltransferase I, are encoded by the *pflA* and *pflB* genes, respectively. PFL activity can be effectively reduced by disruption of either gene [39]. Since we observed 0.4 g/L formate production in the ES07 strain, we inactivated the *pflB* gene to generate strain ES08 (Table 1), and compared the succinate productivity of these two strains. As shown in Table 2, ES08 produced 9.2 g/L succinate with 0.2 g/L formate, resulting in a 63.4% succinate yield. Even though formate formation was ~50% in ES08 compared to ES07, the succinate titer and yield remained the same. Thus, inactivation of PflB slightly reduces formate formation, but does not affect succinate production.

### Inactivation of the glucose-PTS in the ES08 strain

To synthesize succinate via Pathway C (Figure 2), we used strain ES08 to construct a derivative that takes up glucose via a non-PTS mechanism. This alternative mechanism involves *galP*, which encodes galactose/proton symporter (GalP), and *glK*, which encodes GLK. GalP also mediates glucose uptake in PTS-deficient strains of *E. coli* [31]. The *galP* (Genomic ID: CP002824.1, locus tag: EAE_02235) and *glk* (locus tag: EAE_00265) genes are conserved in *E. aerogenes*, and the predicted amino acid sequences are very similar to those of *E. coli* (94 and 91% identity with *E. coli* GalP and GLK, respectively). We therefore speculated that the *E. aerogenes* non-PTS mechanism for glucose uptake occurs in the same manner as in *E. coli*. For inactivation of the glucose-PTS, we disrupted the *ptsG* gene, which encodes the glucose permease enzyme, IIBC^Glc^; we also disrupted the *ptsI* gene that encodes EI, the first enzyme in the PTS phosphorylation cascade (Figure 2). The Δ*ptsG* and Δ*ptsI* mutants were derived from the ES08 strain, and designated as strains ES08Δ*ptsG* and ES08Δ*ptsI*, respectively. Whereas the ES08Δ*ptsG* strain exhibited almost the same growth and colony formation rates as the ES08 strain, the ES08Δ*ptsI* strain grew more slowly and formed much smaller colonies on LB plate.

To evaluate the impact of glucose-PTS deficiency on succinate production, succinate fermentation was carried out using the ES08, ES08Δ*ptsG*, and ES08Δ*ptsI* strains. As shown in Table 2, glucose consumption in the ES08Δ*ptsG* and ES08Δ*ptsI* strains was 11.5 and 6.2 g/L, which is equivalent to 79.3 and 42.8% of that observed in the ES08 strain (14.5 g/L), respectively. The succinate titer in the ES08Δ*ptsG* (8.8 g/L) and ES08Δ*ptsI* (5.4 g/L) strains was less than that in the ES08 strain (9.2 g/L), whereas the succinate yield in the ES08Δ*ptsG* (76.5%) and ES08Δ*ptsI* (87.1%) strains was higher than that recorded in the ES08 strain (63.4%). The titer of pyruvate, which is a main by-product formed in the ES08 strain (1.0 g/L), was lower in the ES08Δ*ptsG* (0.6 g/L) and ES08Δ*ptsI* (0.3 g/L) strains (Table 2). Thus, we succeeded to repress pyruvate formation derived from glucose-PTS. Inactivation of the glucose-PTS leads to a reduced succinate titer, but increases succinate yield in the ES08 strain.

### Succinate fermentation in the ES08Δ*ptsG* strain at pH 5.7-controlled conditions

**Figure 5:**
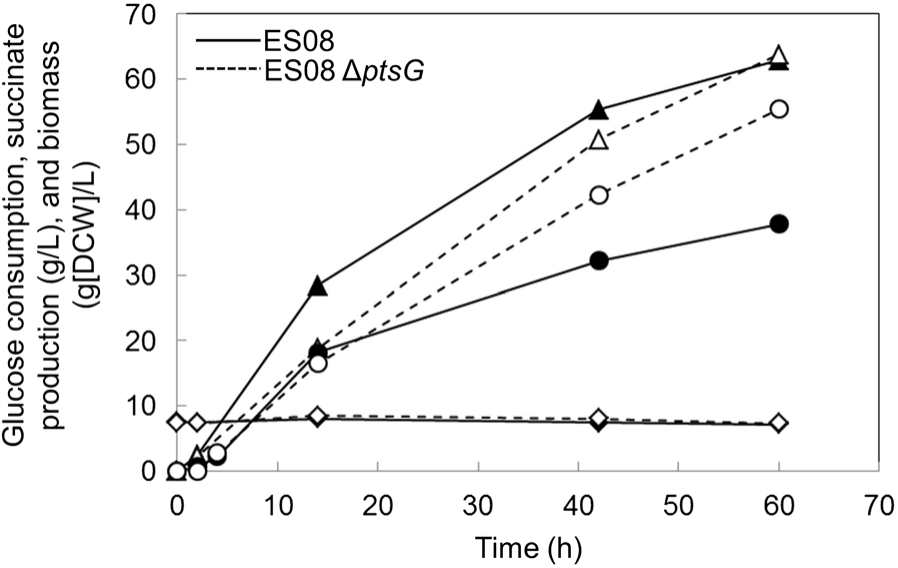
Typical profiles of succinate production, glucose consumption, and biomass in ES08 and ES08Δ*ptsG* strains when cultured at pH 5.7. *Symbols* indicate succinate produced by the ES08 strain (*closed circles*) and the ES08Δ*ptsG* strain (*open circles*), glucose consumption of the ES08 strain (*closed triangles*), and the ES08Δ*ptsG* strain (*open triangles*), and biomass of the ES08 strain (*closed diamond*) and the ES08Δ*ptsG* strain (*open diamonds*).

A “dual-phase” succinate production process, which comprises an initial aerobic cell-growth phase followed by an anaerobic succinate-production phase, has been widely studied in *E. coli* [40]. In addition to the low succinate production rate in ES08Δ*ptsI* under anaerobic conditions (Table 2), this strain exhibited a slow growth phenotype when it aerobically assimilated glucose. This glucose assimilation deficiency is a disadvantage in terms of both biomass formation in the aerobic phase and succinate productivity in the anaerobic phase. Therefore, we selected the ES08Δ*ptsG* strain and evaluated its succinate productivity under pH-controlled conditions. A 100-mL jar fermentation was carried out using the ES08Δ*ptsG* strain and ES08 as a reference strain at pH 5.7. Initial biomass was adjusted to approximately 7.0 g of dry cell weight per liter (g[DCW]/L). The results are summarized in Table 3 and Figure 5. During the early stage of fermentation, at 14 h, glucose consumption of the ES08Δ*ptsG* strain was 18.8 g/L and approximately 0.7-fold lower than that of ES08 strain (28.4 g/L) (Figure 5). Thereafter, the glucose consumption rate of the ES08 strain gradually decreased, whereas that of the ES08Δ*ptsG* strain was maintained during fermentation. At the conclusion of fermentation (60 h), the ES08 and ES08Δ*ptsG* strains had consumed approximately the same amount of glucose (Table 3). The trends of the succinate titer of the ES08 and ES08Δ*ptsG* strains were similar to those observed for glucose consumption. Specifically, the succinate titer of the ES08Δ*ptsG* strain at 14 h was 16.5 g/L, which was 0.9-fold lower than that of the ES08 strain (18.2 g/L). However, succinate titer and yield in the ES08Δ*ptsG* strain at 60 h were 55.4 g/L and 86.8%, respectively. These values were 1.5- and 1.4-fold higher than those in the ES08 strain (37.8 g/L corresponding to a 60.1% succinate yield), respectively.

**Table 3:** Parameter profiles in reactions with ES08 and ES08Δ*ptsG* at pH 5.7 culture conditions

Strain	Biomass^a^, g[DCW]/L	Consumed glucose, g/L	End-product, g/L						Succinate yield^b^, % (g/g)	Volumetric productivity^c^ (g/L/h)
			Pyruvate	Lactate	Malate	Succinate	Formate	Acetate		
ES08^d^	7.2	62.9	1.0	0.6	0.3	37.8	0.1	1.0	60.1	0.63
ES08Δ*ptsG*	7.3	63.8	<0.1	<0.1	0.1	55.4	0.1	1.8	86.8	0.92

^a^Initial biomass was adjusted to ~7.0 g[DCW]/L.

^b^Succinate yield is grams of product per grams of consumed glucose expressed as a percentage.

^c^Volumetric productivity indicates succinate titer (g/L) per hour (h).

^d^ES08 and ES08Δ*ptsG* strains were cultivated for 60 h.

## Discussion

Lowering culture pH in bacterium-based succinate fermentation is one of the major challenges that must be overcome in order to reduce total production costs and save resources. To realize this, metabolic engineering approaches that focus on optimizing energy balances may be required. This is because anaerobic succinate fermentation under acidic conditions runs a high risk of triggering energy starvation [23]. As shown in Figure 1, succinate synthesis includes carboxylation reactions as PEP or pyruvate is converted to OAA. These reactions are generally catalyzed by PPC or PYC in an energy-dependent manner, which explains why their reactions are associated with a net reduction of the ATP level. Furthermore, the energy requirement of putative succinate exporters in *E. aerogenes* would still further reduce the estimated net ATP yield. In addition to this low energy-acquisition problem during anaerobic succinate synthesis, reducing the culture pH stimulates energy consumption; this is required to drive the proton efflux pump and other acid resistance mechanisms in order to maintain pH homeostasis [15, 16, 23].

To overcome this energy problem, we introduced energy conserving succinate pathway into ES04, resulting in generation of the ES08Δ*ptsG* strain [24, 25]. At pH 5.7 controlled conditions, volumetric productivity and yield in this strain were 0.92 (g/L/h) and 86.8% (Table 3). These values represent 4.0- and 1.2-fold increases over our previously achieved values in the ES04/PYC+PCK strain [21]. Thus, optimizing energy-balance in metabolically engineered *E. aerogenes* was required in order to increase succinate production under weakly acidic conditions. As shown in Figure 5, an increase in net ATP yield caused by disruption of the *ptsG* gene has a positive impact on the rate of succinate production. For example, while succinate production in the ES08 strain gradually decreased over time, the rate was maintained in the ES08Δ*ptsG* strain until the end of cultivation (Figure 5). Finally, the succinate titer in the ES08Δ*ptsG* strain (55.4 g/L) was approximately 1.5-fold higher than that of the ES08 strain (37.8 g/L). We speculated that after 14 h cultivation when succinate concentration is over 15 g/L, the demands of energy used for pH homeostasis and succinate efflux will be increased. In this situation, the ES08Δ*ptsG* strain, which harbors an energy-conserving succinate pathway, is able to avoid energy starvation, thus resulting in higher levels of succinate accumulation compared to the ES08 strain. To more precisely define the relationship between intracellular energy level and succinate titer under these conditions, the intracellular ATP level in these two strains should also be monitored.

Interestingly, the carbon recovery rate in ES08Δ*ptsG* was 0.91 (C-mol/C-mol), which was an increased compared to the parental strain ES08 (0.66). It was suggested that inactivation of the glucose-PTS in ES08Δ*ptsG* contributes to the reduction in intracellular pyruvate levels, and therefore reduces the amounts of pyruvate derivatives, which we were unable to detect. Previous studies have shown that *E. aerogenes* anaerobically produced an excess of carbon dioxide together with hydrogen via the formate pathway [37, 41]. We speculate that elimination of pyruvate node pathways such as those for ethanol, lactate, acetate, and 2,3-butadiol synthesis in the ES08 strain is responsible for an increased flux of carbon toward the remaining metabolic pathways such as the formate pathway, which is controlled by PFL and FHL (Figure 1). Disruption of the *pflB* gene allowed us to successfully reduce formate production, although a basal level still remained (Table 2). These data suggest that carbon dioxide formation via the formate pathway probably engenders a reduced succinate yield in our strains (Figure 1). To minimalize carbon loss, complete elimination of formate production by further deletion of *pflA* will be necessary [39].

To the best of our knowledge, this study is the first to report the phenotype of glucose-PTS-deficient mutants in *E. aerogenes*. Glucose consumption in the ES08Δ*ptsG* strain was only 20% lower than the parental strain ES08 (Table 2), and even the ES08Δ*ptsI* strain maintained glucose consumption at ~40% the level observed in ES08. These results indicate that the glucose-PTS does not serve as the dominant glucose uptake mechanism under anaerobic conditions in *E. aerogenes*. Presumably, other phosphotransferases, such as the fructose-specific IIBC component (IIBC^Fru^) and non-PTS permeases such as GalP, also mediate glucose import under anaerobic conditions. Construction of an ES08Δ*ptsI* Δ *galP* strain, and evaluation of its glucose consumption under these conditions will further elucidate the elements of the *E. aerogenes* anaerobic glucose import system.

From the perspective of theoretical net ATP production and obtained succinate yields, disruption of the *ptsI* gene, rather than *ptsG* deletion, is a more desirable phenotype. Indeed, the best succinate yield (87.1%) we achieved in this study was observed in the ES08Δ*ptsI* strain, in which glucose uptake via the PTS was completely blocked (Figure 1; Table 2). Thus, shifting glucose uptake from the PTS to a non-PTS mechanism leads to increased succinate yield in PCK overexpressed strain, albeit with a reduced succinate titer. This glucose assimilation-deficient phenotype of the ES08Δ*ptsI* strain is a disadvantage with regard to succinate productivity (g/L/h). A previous study has shown that the *E. coli* Δ*ptsI* mutant exhibited a slow growth phenotype during its assimilation of glucose, and that co-expression of *galP* and *glk* restores the glucose assimilation in this strain [31]. In a similar way, we will construct an ES08Δ*ptsI* strain that has simultaneously enhanced GalP and Glk activity, and then evaluate the impact on succinate production under weakly acidic and anaerobic conditions. These studies will significantly improve bacterium-based succinate fermentation under acidic and anaerobic conditions.

## Conclusions

To achieve improved succinate production under weakly acidic and anaerobic conditions, we introduced an energy-conserving succinate pathway into *E. aerogenes*, and then generated the ES08Δ*ptsG* strain. This strain produced 55.4 g/L of succinate from glucose, with an 87.1% yield after 60 h of culture at pH 5.7 and anaerobic conditions. Thus, we highlight the potential of bacterium-based succinate fermentation using recombinant *E. aerogenes* under anaerobic and weakly acidic conditions.

## Methods

### Bacterial strains, plasmids, and culture conditions

The strains and plasmids used in this study are summarized in Table 1. *E. aerogenes* AJ110637 was deposited at the International Patent Organism Depository, Agency of Industrial Science and Technology (Japan), under accession number FERM P-21348, and then assigned an international deposit number FERM BP-10955 [42]. Plasmids were introduced into *E. coli* and *E. aerogenes* by electro-transformation. Both *E. coli* and *E. aerogenes* were grown in Luria-Bertani (LB) medium at 37°C. When needed, 50 mg/L kanamycin or 40 mg/L chloramphenicol were added to select transformants and to maintain the plasmids. The strains harboring pSTV28-*pck* are indicated by the strain name following by ‘PCK’ (e.g., the ES04 strain harboring pSTV28-*pck* is designated as ES04/PCK).

### Disruption of the *poxB*, *pflB*, *ptsG*, and *ptsI* genes

To disrupt the *poxB*, *pflB*, *ptsG*, and *ptsI* genes on the *E. aerogenes* chromosome, the λ Red-dependent recombination system was used with the Red-recombineering helper plasmid pRSFRedTER [43, 44]. A removable kanamycin resistance gene flanked by *attL*_λ_ and *attR*_λ_ was amplified with Δ*poxB*-*attL*/Δ*poxB*-*attR*, Δ*pflB*-*attL*/Δ*pflB*-*attR*, Δ*ptsG*-*attL*/Δ*ptsG*-*attR*, and Δ*ptsI*-*attL*/Δ*ptsI*-*attR* primers containing 60-nt sequences homologous to the target region at the 5′-end of the chromosome. The pMW-*attL*_*λ*_-Km^R^-*attR*_*λ*_ plasmid was used as the DNA template [44, 45]. Replacement of the target genes on the chromosome with the *attL*_*λ*_-Km^R^-*attR*_*λ*_ fragment was confirmed by PCR using Δ*poxB*-*CF*/Δ*poxB*-*CR*, Δ*pflB*-*CF*/Δ*pflB*-*CR*, Δ*ptsG*-*CF*/Δ*ptsG*-*CR*, and Δ*ptsI*-*CF*/Δ*ptsI*-*CR* as primers. To remove pRSFRedTER from the marker strains, the strains were spread on LB plates containing 10% sucrose and 1 mM isopropyl β-d-1-thiogalactopyranoside to obtain single colonies. All primer sequences are listed in Table 4.

**Table 4:** The sequences of the oligonucleotide primers used in this study

Primer	Primer sequences (5′–3′)
Disruption of *poxB*, *pflB*, *ptsG*, *and ptsI* genes	
Δ*poxB*-*attL*	5′-ACGTAACCTGTAGTTTCATCTAAGCTTGATAGCGTTATCACAAAAAGGAGATGGAAAACCTGAAGCCTGCTTTTTTATACTAAGTTGGC-3′
Δ*poxB*-*attR*	5′-TATCACTGCGAAGATCTATCACGGCATATCCTTGTTCTGATTCAGGGTGATAGCATTGTTTACTCAGCCTTATTTTTCAGATTTTATTCGG-3′
Δ*pflB*-*attL*	5′-CATTAATGGTTGTCTCAGGCAGTAAATAAAAAATCCACTTAAGAAGGTAGGTGT TACATGTGAAGCCTGCTTTTTTATACTAAGTTGGC-3′
Δ*pflB*-*attR*	5′-AAAAAGGCCCCACTGATGTGGGGCCTTTATTGTACGCTTTTTCAGTCAGACAG GGAATTACGCTCAAGTTAGTATAAAAAAGCTGAACGA-3′
Δ*ptsG*-*attL*	5′-ATGTTTAAGAATGCATTTGCTAACCTGCAGAAGGTCGGTAAATCGCTGATGCTGCCAGTATGAAGCCTGCTTTTTTATACTAAGTTGGC-3′
Δ*ptsG*-*attR*	5′-TTAGCTATTGCGGATGTACTCATCCATCTCGGTTTTCAGGTTATCGGACTTGGTGCCGAACGCTCAAGTTAGTATAAAAAAGCTGAACGAG-3′
Δ*ptsI*-*attL*	5′-TGAACTCGAGTAAGTTCACGGGTTCTTTTTAATATCAGTCACAAGTAAGGTAGGGTTATGTGAAGCCTGCTTTTTTATACTAAGTTGGC-3′
Δ*ptsI*-*attR*	5′-ATCGAACAAACCCATGATCTTCTCCTAAGCAGTAATTTGGGCCGCGCATCTCGTGGATTACGCTCAAGTTAGTATAAAAAAGCTGAACGA-3′
Δ*poxB*-*CF*	5′-GTCGATTAGCTTATGTAGACCG-3′
Δ*poxB*-*CR*	5′-TGGAGTTCATTGACGGTTGGG-3′
Δ*pflB*-*CF*	5′-GGCTTTGAACACAGCATCGC-3′
Δ*pflB*-*CR*	5′-CTGTTTTGACAGGTACCC-3′
Δ*ptsG*-*CF*	5′-TGAATGAAACGTGATAGCCG-3′
Δ*ptsG*-*CR*	5′-TCAACTATAGACTTAGCTGC-3′
Δ*ptsI*-CF	5′-CAAGAAGTTACCATTACCGC-3′
Δ*ptsI*-CR	5′-TTGCCAGTAGGTTTGATGGC-3′
*frdABCD*-*CF*	5′-GCGGCGATATTACATCCTGA-3′
*frdABCD*-*CR*	5′-TCTAAGTGTAGATAGCGGCA-3′
*A. succinogenes* PCK expression cassette	
*pck*-*tacR*	5′-CGAGTTTGTTTAAGTCAGTCATGGCAGTCTCCTTGTGTGAAATTGTTATCCG-3′
*tac*-*pckF*	5′-CGGATAACAATTTCACACAAGGAGACTGCCATGACTGACTTAAACAAACTCG-3′
Δ*poxB*-*pckR*	5′-TATCACTGCGAAGATCTATCACGGCATATCCTTGTTCTGATTCAGGGTGATAGCATTGTTATCTCAGCCTTATTTTTCAGATTTTATTCGG-3′
Promoter replacement of *frdABCD*	
*frdABCD*-*tacF*	5′-AAATAAAATTGATAAATTAGCGCACGGATTGATAAAAAAATCGAACGCGTTGAAGCCTGCTTTTTTATACTAAGTTGGCA-3′
*frdABCD*-*tacR*	5′-CCTGCTCCGCCTGCGCCAATGACGACAAGATCGGCTTGAAAAGTTTGCACGGCAGTCTCCTTGTGTGAAATTGTTATCCG-3′

### Removal of the kanamycin resistance gene

To remove the kanamycin resistance gene on the chromosome, the λ Int/Xis-dependent excision system was used with the λ Int/Xis expression plasmid pRSF-*P*_*ara*_-IX [21]. Transformants harboring pRSF-*P*_*ara*_-IX were selected on LB plates containing 40 mg/L of chloramphenicol. Obtained transformants were spread on LB plates containing 40 mg/L chloramphenicol and 1% l-arabinose. Clones that formed single colonies were identified and elimination of the kanamycin resistance gene was confirmed by growth on LB medium containing 50 mg/L kanamycin. Strains that could not grow on the kanamycin plates were utilized as marker-free strains. The procedure to remove pRSF-*P*_*ara*_-IX from the marker-free strain was the same as that used with pRSFRedTER.

### Construction of the ES06 strain containing an integrated *A. succinogenes* PCK expression cassette at the *poxB* gene locus

Schemes of the integration of an *A. succinogenes* PCK expression cassette are summarized in Figure 3a DNA fragment containing a removable kanamycin resistance gene and *tac* promoter region was amplified with Δ*poxB*-*attL* (primer 1) and *pck*-*tacR* (primer 2) primers using *attL*_*λ*_-Km^R^-*attR*_*λ*_-*P*_*tac*_ as a template (Figure 3a) [45]. Primers *tac*-*pckF* (primer 3) and Δ*poxB*-*pckR* (primer 4) were designed based on the genome sequence of the *A. succinogenespck* gene (Genomic ID: CP000746 Locus lag; Asuc_0221). PCR was performed using genomic DNA from the *A. succinogenes* ATCC55618 as a template to obtain the *pck* gene fragment (Figure 3a). Overlapping PCR was then performed using Δ*poxB*-*attL* (primer 1) and Δ*poxB*-*pckR* (primer 4) primers with both of the above PCR products as templates in order to obtain the *A. succinogenes* PCK expression cassette. This cassette, which includes a kanamycin resistance gene and *A. succinogenespck* gene under the control of *tac* promoter, was introduced into an ES04 strain harboring pRSFRedTER by using the λ Red-dependent recombination system at the locus of the *poxB* gene (Figure 3b). Replacement of the *poxB* gene on the chromosome with the cassette was confirmed by PCR using Δ*poxB*-*CF* (primer 5)/Δ*poxB*-*CR* (primer 6) (Figure 3c). The obtained strain was designated as the ES06 strain (Table 1). The kanamycin resistance gene was removed by the λ Int/Xis-dependent excision system using pRSF-*P*_*ara*_-IX (Figure 3d).

### SDS-PAGE analysis

Samples were obtained from cells grown on LB plates at 37°C for 10 h. The populations were harvested by centrifugation at 21,600×*g* for 1 min at 4°C. The harvested cells were washed three times with 0.1 M Tris–HCl (pH 7.0) buffer. The washed cells were re-suspended and sonicated for 20 min (20 s treatment was repeated with 20-s intervals) at 4°C with a Bioruptor UCD-250 (Cosmo Bio Co. Ltd.; Tokyo, Japan). The disrupted cells were centrifuged at 21,600×*g* for 10 min at 4°C to remove cell debris and then subjected to ultracentrifugation at 166,000×*g* for 60 min at 4°C to remove the insoluble fraction. The soluble fraction was used in SDS-PAGE analysis using the NuPAGE SDS-PAGE Gel system (Invitrogen). Proteins were separated by electrophoresis using 4–12% Bis–Tris NuPAGE gels with MES running buffer, and then stained with Coomassie Brilliant Blue. Protein concentration in the soluble fraction was determined using the Pierce 660 nm Protein Assay Kit (Thermo Fisher Scientific Inc.; Waltham, MA, USA). The molecular weight marker was Novex^™^ Sharp Unstained Protein Standard (Invitrogen).

### Replacement of the promoter at the *frdABCD* locus

To replace the promoter region of *frdABCD* genes with the *tac* promoter, the λ Red-dependent recombination system was used. A DNA fragment was amplified using *frdABCD*-*tacF*/*frdABCD*-*tacR* primers and *attL*_*λ*_-Km^R^-*attR*_*λ*_-*P*_*tac*_ as a template [45]. Replacement of the promoter region at the *frdABCD* locus with the *attL*_*λ*_-Km^R^-*attR*_*λ*_-*P*_*tac*_ fragment was confirmed by PCR using *frdABCD*-*CF*/*frdABCD*-*CR* primers.

### Succinate fermentation using 1.5-mL microfuge tubes

To evaluate succinate production under weakly acidic (pH < 6.2) and anaerobic conditions, we used a convenient evaluation system with 1.5-mL microfuge tubes [20]. Pre-culturing for cell growth was performed on LB plates at 37°C for 10 h. The pre-cultured cells were then incubated anaerobically at 37°C for 16 h in an AnaeroPack A-04 (Mitsubishi Gas Chemicals Inc.; Tokyo, Japan), and inoculated into 1.5-mL microfuge tubes with 1.4 mL MS medium [40 g/L glucose, 1 g/L MgSO_4_·7H_2_O, 2 g/L Bacto yeast extract, 1 g/L (NH_4_)_2_SO_4_·7H_2_O, 1 g/L KH_2_PO_4_, 10 mg/L MnSO_4_·5H_2_O, 10 mg/L FeSO_4_·7H_2_O, and 1 mg/L biotin] containing 50 g/L precipitated CaCO_3_ sterilized by dry heat at 180°C for 3 h (Japanese Pharmacopoeia; Tokyo, Japan). After tightly capping the tubes, succinate fermentation was performed using an Eppendorf Thermomixer comfort (Eppendorf; Hamburg, Germany) at 34°C and a rotation speed of 1,400 rpm. Initial biomass was adjusted to approximately 5.0 g[DCW]/L. Using this system, there was a drop in pH from 6.2 to ~5.4 due to the formation of acidic compounds such as succinate during fermentation.

### pH-controlled succinate fermentation using a 100-mL jar fermenter

pH-controlled succinate fermentation was carried out in a 100-mL jar fermenter with 60 mL MS3 medium [90 g/L glucose, 1 g/L MgSO_4_·7H_2_O, 2 g/L Bacto yeast extract, 1 g/L (NH_4_)_2_SO_4_·7H_2_O, 1 g/L KH_2_PO_4_, 10 mg/L MnSO_4_·5H_2_O, 10 mg/L FeSO_4_·7H_2_O, 1 mg/L biotin, and 0.05 g GD113 (antifoam reagent)]. The initial pH was adjusted to 5.7 using Ca(OH)_2_ at 34°C. pH was maintained with 4 N Ca(OH)_2_ during cultivation. Carbon dioxide gas was applied at 40 mL/min and agitation was set at 700 rpm. Pre-culture was performed on LB agar at 37°C for 10 h. The cells were then incubated at 37°C for 16 h in an AnaeroPack A-04. Initial biomass was adjusted to approximately 7.0 g[DCW]/L.

### Analysis of metabolites

Organic acids that accumulated in the medium were analyzed using high-performance liquid chromatography on a CDD-10AD system (Shimadzu Co. Ltd.; Kyoto, Japan) after a suitable dilution [46]. When the broth contained crystallized succinate calcium, it was diluted with 0.1 N HCl before measuring. The glucose concentration was analyzed using an AS-310 Biotech Analyzer (Sakura SI Co. Ltd.; Tokyo, Japan). Yield is expressed as gram of product per gram of consumed glucose (g/g). Optical densities (OD) were measured at 600 nm with a U-2001 spectrometer (Hitachi Co. Ltd.; Tokyo, Japan). The broth containing CaCO_3_ was diluted with 0.1 N HCl before measuring OD_600_. Dry cell weight was calculated according to a formula: g[DCW]/L = 0.291 × OD_600_.

## Abbreviations

ATP: adenosine triphosphate
DCW: dry cell weight
FHL: formate hydrogen lyase
FRD: fumarate reductase
FUM: fumarase
GalP: galactose/proton symporter
GLK: glucokinase
Int: Lambda integrase
LB: Luria-Bertani
MES: 2[*N*-Morpholino] ethanesulfonic acid
MDH: malate dehydrogenase
OAA: oxaloacetate
OD: optical density
PBS: polybutylene succinate polymer
PCK: phosphoenolpyruvate carboxykinase
PEP: phosphoenolpyruvate
PFL: pyruvate formate lyase
POX: pyruvate oxidase
PPC: phosphoenolpyruvate carboxylase
PTS: phosphotransferase system
PYC: pyruvate carboxylase
Xis: Lambda excisionase
